# Machine learning on asynchronous clinical pages to predict clinical deterioration

**DOI:** 10.1093/jamiaopen/ooag122

**Published:** 2026-07-17

**Authors:** Isabel C Arvelo, Kipp Shipley, Adam Wright, Bryan D Steitz

**Affiliations:** Data Science Institute, Vanderbilt University, Nashville, TN 37212, United States; Department of Anesthesiology, Vanderbilt University Medical Center, Nashville, TN 37203, United States; Department of Biomedical Informatics, Vanderbilt University Medical Center, Nashville, TN 37203, United States; Department of Biomedical Informatics, Vanderbilt University Medical Center, Nashville, TN 37203, United States

**Keywords:** clinical deterioration, early warning score, asynchronous communication, deep learning

## Abstract

**Objectives:**

Clinical deterioration in hospitalized patients is often preventable, but traditional early warning scores based on structured data are limited by delayed or inconsistent documentation. We developed and evaluated a machine learning pipeline that predicts clinical deterioration using real-time pager messages exchanged between clinicians.

**Materials and Methods:**

We conducted a retrospective study of adult non-ICU hospitalizations at Vanderbilt University Medical Center between January 2018 and June 2021. Using the content and frequency of messages, we trained long short-term memory models to predict rapid response activation, unplanned ICU transfer, or cardiac arrest within the next 6, 12, or 24 hours. Model performance was compared with the Epic’s Deterioration Index and with a logistic regression ensemble combining predictions from both models.

**Results:**

There were 1 519 445 pages associated with 111 346 hospitalizations among 74 912 patients. Deterioration events were observed in 5114 (4.6%) hospitalizations. The model achieved moderate discriminative ability, with areas under the receiver operating characteristic curve (AUROC) of 0.684 (95% CI, 0.66-0.71), 0.724 (95% CI, 0.71-0.74), and 0.669 (95% CI, 0.65-0.68) for predicting deterioration within 6, 12, and 24 h, respectively. Predictions showed moderate correlation with EDI scores (Pearson’s R, 0.297-0.323) suggesting complementary signals. The ensemble model consistently outperformed either approach alone, achieving an AUROC of 0.797 (95% CI, 0.78-0.82) for 12-h prediction.

**Discussion and Conclusion:**

Clinical pages represent an underutilized data source capturing clinicians’ intuition and observations before they appear in formal documentation. Machine learning on pages can augment early warning systems by providing real-time information and intuition without increasing workload.

## Background and significance

Unexpected clinical deterioration in hospitalized patients is a critical patient safety concern that can lead to preventable cardiac arrests, unplanned intensive care unit (ICU) transfers, and death. Patients commonly exhibit signs of deterioration hours before serious events,^1^^,^^2^ but healthcare organizations often struggle to detect and respond effectively.^3^ Many hospitals have implemented rapid response systems, which combine an afferent limb for detecting at-risk patients with an efferent limb, such as rapid response teams (RRTs), that deliver timely interventions.^4^^,^^5^ When deployed effectively, these systems can reduce unplanned ICU transfers, cardiopulmonary arrests, and hospital length of stay.^1^^,^^6^

Early warning scores (EWS) are commonly used to identify patients at risk for clinical deterioration by monitoring structured lab or vital sign data from the electronic health record (EHR). However, these data are frequently delayed or inconsistently documented,^7–9^ which contributes to poor performance in detecting imminent events.^10–12^ In adult inpatient populations, commonly implemented EWS demonstrate only moderate discrimination (area under the receiver operating characteristic curve [AUROC] 0.62-0.78) and often detect fewer than 25% of events,^13–15^ though reported performance varies widely across institutions and care settings.^16–18^ Clinicians often recognize subtle signs of deterioration before they are reflected in early warning scores,^19^^,^^20^ and incorporating intuition into EWS has been shown to improve performance.^13^^,^^21^ Many rapid response systems allow activation based on clinical concern, but barriers such as uncertainty or lack of confidence can hinder escalation of care.^22^

Few EWS incorporate clinical judgment through structured assessments or score modifications,^23^ but distilling intuition and worry into a structured variable may miss important nuances in patient condition. Documentation of concern is often captured in free-text notes or comments, which may not reflect a patient’s current or evolving condition.^24–26^ Many healthcare organizations use pager messages for real-time communication of patient needs and clinical concerns.^27^ These messages, exchanged among multiple clinicians throughout a hospitalization, provide a unique window into patient status by capturing observations not apparent in structured documentation. Prior work has shown that paging data contain valuable information about patient conditions^28^^,^^29^ and can reflect clinical worry and intuition.^13^ In this study, we examined whether machine learning models trained on the content and frequency of pager messages could identify patients at risk of imminent deterioration.

## Methods

This study was conducted at Vanderbilt University Medical Center (VUMC), a large academic medical center in middle Tennessee which includes a 1051-bed adult hospital.^30^ VUMC uses an Epic EHR (Epic Systems Corporation) for all clinical functions. Clinicians at VUMC use unidirectional text-based electronic pages as a primary form of communication among hospital teams. Epic Secure Chat is not currently implemented at VUMC.

This study was approved and granted a waiver of consent by Vanderbilt University Institutional Review Board. We followed the Transparent Reporting of a Multivariable Prediction Model for Individual Prognosis or Diagnosis (TRIPOD) and Minimum Information for Medical AI (MINIMAR) reporting guidelines.^31^

### Study design and population

This retrospective study included all patients who were at least 18 years old at the time of admission and received inpatient care on non-ICU hospital wards at Vanderbilt University Hospital between January 1, 2018, and June 30, 2021. Exclusion criteria included patients hospitalized for less than 1 day, patients receiving palliative care, and patients admitted directly to an intensive care unit (ICU). We labeled each hospitalization for the first of 3 deterioration events: rapid response activation, unplanned ICU transfer, and in-hospital cardiac arrest occurring in hospital wards. All deterioration events were confirmed through manual chart review by an expert clinician (KS) on the VUMC RRT. We defined unplanned ICU transfer as any transfer to intensive care that did not originate from an operating room, procedural area, or emergency department.

### Data collection and preprocessing

**Figure 1.**
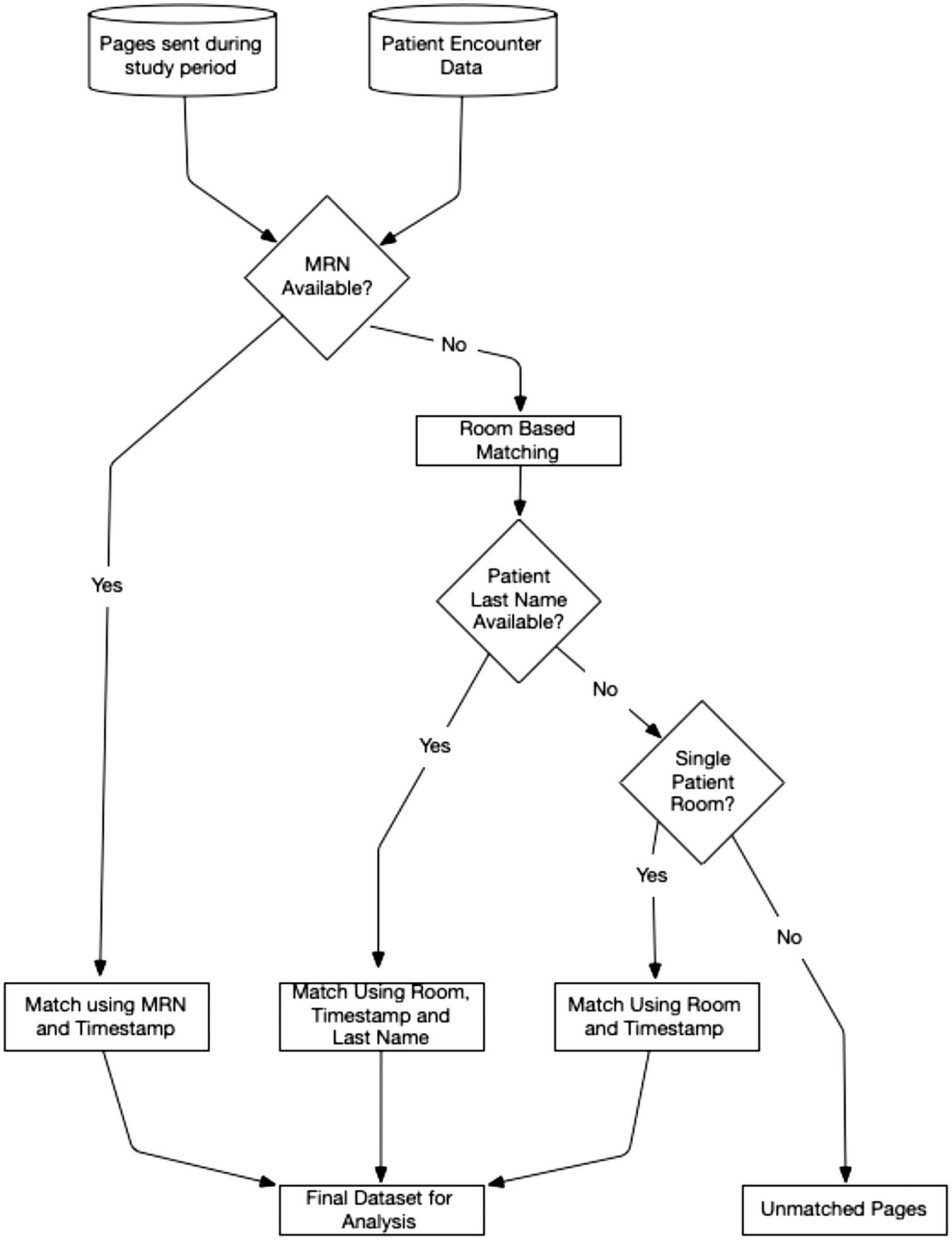
Pipeline for matching care team pages to patient encounters.

From VUMC’s Epic Clarity reporting database, we extracted patient sociodemographic characteristics and hospitalization details. Patient-level socioeconomic data, including insurance status and Area Deprivation Index, were not available in the studied dataset. We also extracted all pages sent about patients in our study. Each page included a unique identifier, page timestamp, patient name, patient room, medical record number (MRN), and message text. We associated pages to patient encounters by matching MRN and timestamp (Figure 1). In cases where the MRN was not available, we mapped the page to encounter using patient name, room, and timestamp. Overall, 22.5% of pages could not be linked to a specific encounter and were excluded from our study. Our manual review of unmatched pages determined that these pages communicated non-clinical information including scheduling, room availability, and bed management. A random sample of deidentified example pages is presented in Appendix A.

### Feature selection

Model features representing each page included word embeddings, patient age (years), and minutes since admission. Word embeddings are numerical vectors that represent the semantic meaning of text. Embeddings were generated using Clinical BERT (Bidirectional Encoder Representations from Transformers),^32–34^ which has been shown to produce contextually rich representations of medical language.^33^^,^^35^ We performed domain adaptation of Clinical BERT on the corpus of pages using masked language modeling (MLM) to adapt the model to our institution’s specific context. For each page, we used the final hidden-layer embedding of the [CLS] token as a fixed-length representation of the message.

**Figure 2.**
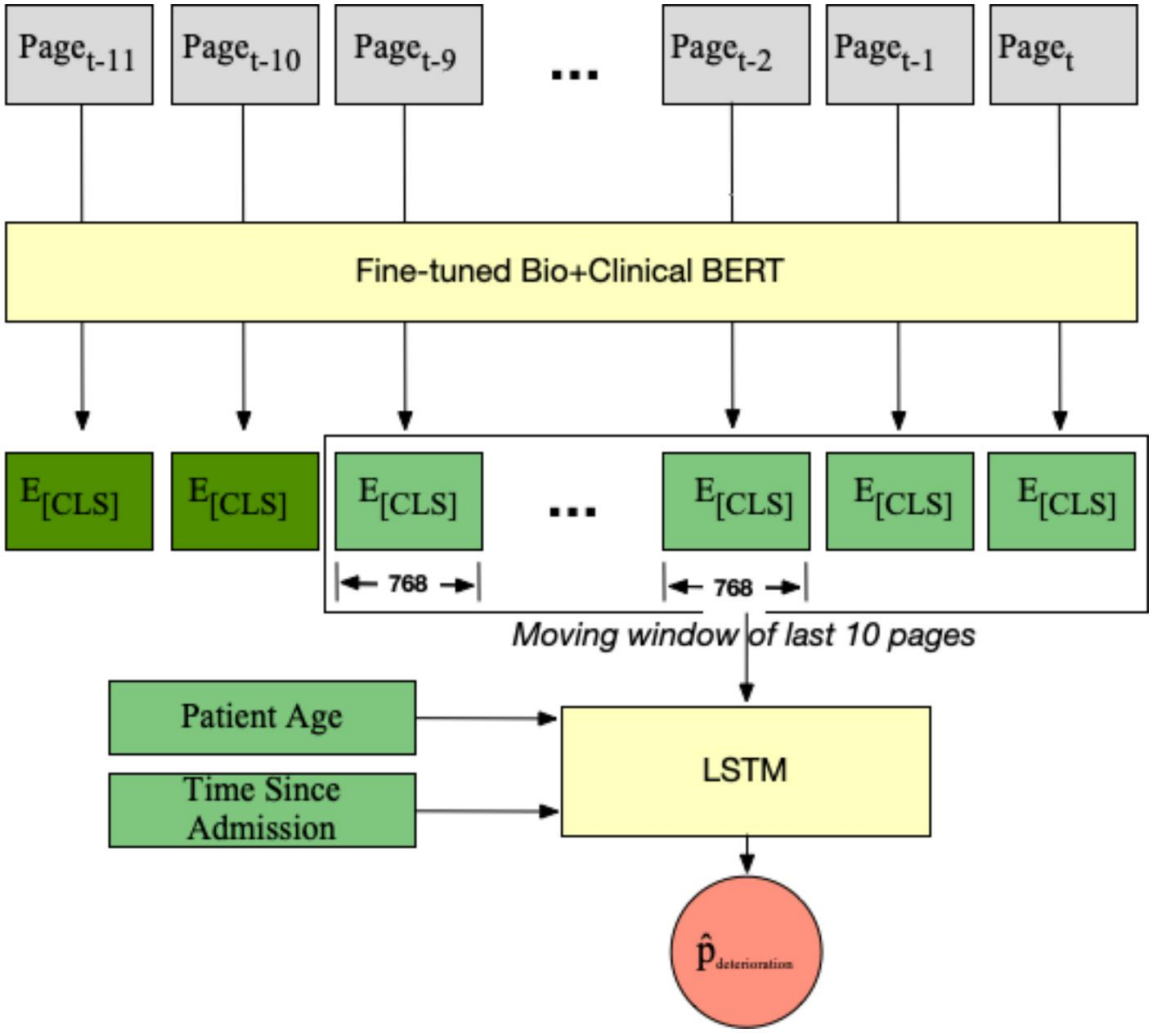
Machine learning pipeline.

We aggregated page-level features, per encounter, for input into our models (Figure 2). We applied a sliding window approach to evaluate the 10 most recent pages per encounter. During model development, we evaluated several window sizes (ranging from 1 to 20 pages) and found that using the 10 most recent pages provided the best overall performance. To reduce information leakage from pager messages that explicitly reference imminent deterioration or escalation, we implemented a 3-hour washout period prior to the first deterioration event.^13^ Pages occurring within this window were excluded from model input to prevent the model from learning trivial cues that directly signal the outcome rather than antecedent clinical concern. For encounters without an event, we maintained all pages that met the study criteria. The model begins generating predictions 24 h after admission, excluding the initial period of routine assessments, diagnostic tests, and treatment adjustments, to distinguish expected clinical activities and true signs of deterioration.

### Model development

We trained 2-layer long short-term memory (LSTM) models to predict clinical deterioration. The intended users of the model outputs are Rapid Response and other acute care escalation teams, who would incorporate these predictions as part of their afferent limb response to identify patients at risk of clinical deterioration. The model generates a prediction score for each page to provide real-time indication of potential deterioration. This page-level scoring allows clinicians to focus attention on patients flagged by particularly concerning messages, while clinical judgment determines whether the alert reflects true deterioration. Unlike traditional machine learning models that analyze features at a single point in time, LSTMs learn patterns from sequential data.^36^ Encounter-level data were divided by year into a training set (80%) and a testing set (20%), with 25% of training encounters randomly held out for hyperparameter tuning and early stopping to prevent overfitting. Hyperparameters were optimized using the Hyperband algorithm^37^ with preset hyperparameter ranges (Appendix B). We monitored validation loss to determine early stopping. We trained separate models to predict deterioration across 3 time horizons: 6, 12, and 24 hours. The same training splits were applied across all 3 time-horizons for consistent evaluation. Models were implemented and evaluated using Tensorflow (version 2.11.0), Keras (2.11.0), and scikit-learn (0.24.1) packages in Python 3.8.8. All model training and evaluation were performed using data from VUMC. External validation at another institution was not performed.

### Statistical analysis

We compared characteristics of patients who experienced deterioration events vs those who did not using Welch *t*-tests for numerical features and *χ*^2^ tests for categorical features. We considered a *P* value less than 0.05 to be statistically significant.

We assessed model performance using a holdout test set containing 20% of encounters, all after June 2020, including only encounters with at least one page sent within the evaluated time horizon. Evaluation metrics included area under the receiver-operating characteristic curve (AUROC), area under the precision-recall curve (AUPRC), sensitivity, specificity, F-score, and positive predictive value (PPV). Classification thresholds were optimized to achieve high sensitivity while maintaining clinically meaningful PPV (>10%), consistent with standards in early warning system literature.^38–41^

We compared our paging model to Epic’s Deterioration Index (EDI; score ≥ 60), which has been implemented at VUMC since July 2020 and has demonstrated the best performance among available EWS.^13^ We chose the EDI as our baseline comparator because it is widely implemented and currently represents the standard of care across many organizations, including VUMC. To enable direct comparison, we carried forward predictions from our paging model to align with EDI’s 15-min update intervals.^42^ Performance was evaluated using bootstrapping with 100 iterations. We randomly sampled one observation per encounter in each iteration, then calculated evaluation metrics with 95% confidence intervals. We also evaluated performance at the encounter level, classifying an encounter as a true positive if at least one prediction occurred within the specified time horizon for patients who experienced deterioration.

To assess whether combining information from both models improved prediction, we developed a logistic regression ensemble incorporating paging model predictions, the latest EDI score, time since admission, and time since last prediction. Ensemble hyperparameters were tuned using nested cross-validation with 5 outer folds stratified by outcome and 3-fold inner validation, repeated 50 times with random splits while maintaining both stratification and patient-level grouping. This approach generated out-of-fold predictions for all patients, ensuring that predictions were made using models trained on separate data. We report the mean performance across all iterations along with 95% confidence intervals. The final bootstrap fixed-interval analysis used a single cross-validation repeat to reflect real-world deployment and provide a realistic assessment of performance in clinical practice.

## Results

Between January 2018 and June 2021, 74 912 patients (mean [SD] age, 53.6 [19.0] years; 39 322 (47.5%) women) experienced 111 346 hospitalizations and were the subject of 1 519 445 pages. During each hospitalization, patients were the subject of a median [IQR] of 9 [13] pages. Deterioration events occurred in 5114 (4.6%) hospitalizations, most commonly including rapid response activations (2676 [52.3%]), ICU transfers (1835 [35.9%]), and in-hospital cardiac arrests (603 [11.8%]), at a median [IQR] 4.4 [6.0] days into the admission. Prior to a deterioration event, care teams sent a median [IQR] 6.5 [21] pages, with the last page sent a median [IQR] 6.5 [9.4] h before deterioration. There were 645 (12.6%) deterioration events without pages in the preceding 24 h, 1446 (28.3%) without pages in the preceding 12 h, and 2749 (53.8%) without new pages in the preceding 6 h. Encounter-level demographics are presented in Table 1.

**Table 1.** Encounter-level demographic statistics of study population.

	Encounters, no (%)			
	Deterioration event (*n* = 5114)	No deterioration event (*n* = 106 232)	All encounters (*n* = 111 346)	*P* value
Age (years)				<.001
Mean (SD)	59.58 (16.3)	54.00 (18.54)	54.26 (18.5)	
Median (IQR)	62 (21)	56 (31)	56 (31)	
Sex				<.001
Female	2184 (42.7)	55 089 (51.9)	57 273 (51.4)	
Male	2929 (57.3)	51 139 (48.1)	54 068 (48.6)	
Unknown	1 (0.0)	4 (0.0)	5 (0.0)	
Race				.09
American Indian or Alaska Native	17 (0.3)	336 (0.3)	353 (0.3)	
Asian	12 (0.2)	445 (0.4)	457 (0.4)	
Black or African American	933 (18.2)	18 967 (17.9)	19 900 (17.9)	
Hispanic/Latino/a	19 (0.4)	539 (0.5)	558 (0.5)	
Middle Eastern/North African	3 (0.1)	129 (0.1)	132 (0.1)	
Pacific Islander	0 (0.0)	22 (0.0)	22 (0.1)	
White	3962 (77.5)	81 775 (77.0)	85 737 (77.0)	
Other/unknown	168 (3.3)	4019 (3.8)	4187 (3.8)	
Length of stay (days)				<.001
Mean (SD)	16.47 (15.3)	5.08 (5.4)	5.6 (6.3)	
Median (IQR)	12.04 (13.1)	3.54 (3.7)	3.7 (4.00)	
Average number of pages				<.001
Mean (SD)	21.52 (22.9)	13.27 (15.2)	13.65 (15.8)	
Median (IQR)	14 (21)	9 (13)	9 (13)	

**Figure 3.**
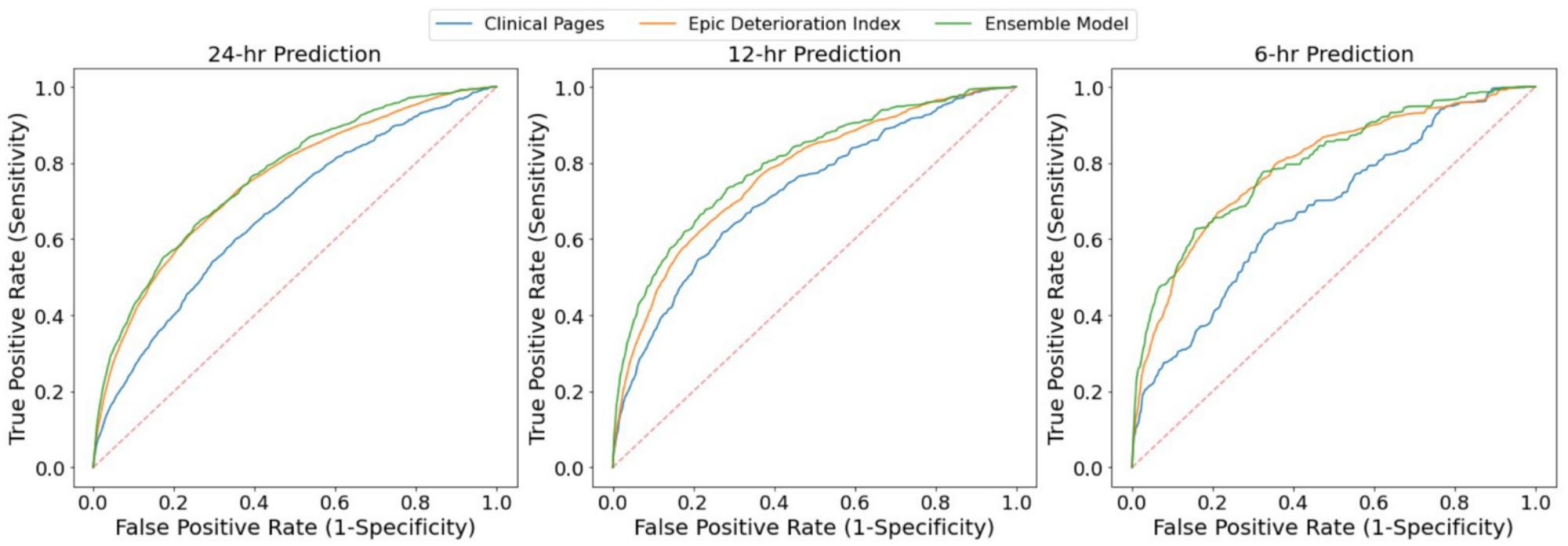
Comparison of model discrimination across prediction windows.

Our model demonstrated varying performance across prediction windows (Table 2; Appendix C). The 12-hour paging model showed the strongest discrimination (AUROC [95% CI], 0.724 [0.707-0.741]; F-score [95% CI], 0.086 [0.068-0.105]) (Figure 3). The EDI outperformed the paging at 6 hours (AUROC [95% CI], 0.786 [0.670-0.892] vs 0.684 [.659-.710]), 12 hours (AUROC [95% CI], 0.773 [0.728-0.814] vs 0.724 [0.707-0.741]), and 24 hours (AUROC [95% CI] 0.752 [0.730-0.771] vs 0.669 [0.654-0.681]) before a deterioration event. Appendix D details encounter-level classification performance.

**Table 2.** Comparison of model performance predicting clinical deterioration in 6-, 12-, and 24 hours.

	Score, no. (95% CI)					
	AUROC	AUPRC	Sensitivity	Specificity	PPV	F-Score
Pages						
6 hours	0.684 (0.659-0.710)	0.022 (0.012-0.038)	0.265 (0.222-0.313)	0.983 (0.982-0.984)	0.061 (0.050-0.072)	0.098 (0.082-0.170)
12 hours	0.724 (0.707-0.741)	0.041 (0.030-0.056)	0.140 (0.107-0.172)	0.980 (0.979-0.982)	0.062 (0.050-0.077)	0.086 (0.068-0.105)
24 hours	0.669 (0.654-0.681)	0.048 (0.039-0.057)	0.152 (0.131-0.173)	0.963 (0.961-0.965)	0.067 (0.057-0.079)	0.093 (0.078-0.109)
EDI^a^						
6 hours	0.786 (0.670-0.892)	0.013 (0.002-0.048)	0.177 (0.046-0.367)	0.981 (0.980-0.983)	0.010 (0.002-0.022)	0.018 (0.005-0.042)
12 hours	0.773 (0.728-0.814)	0.028 (0.016-0.048)	0.170 (0.111-0.251)	0.980 (0.978-0.981)	0.036 (0.025-0.054)	0.064 (0.041-0.088)
24 hours	0.752 (0.730-0.771)	0.052 (0.040-0.068)	0.154 (0.115-0.188)	0.979 (0.978-0.980)	0.082 (0.061-0.103)	0.107 (0.081-0.133)
Ensemble model^b^						
6 hours	0.802 (0.778-0.821)	0.041 (0.030-0.054)	0.265 (0.222-0.313)	0.983 (0.982-0.984)	0.061 (0.050-0.072)	0.098 (0.082-0.117)
12 hours	0.797 (0.777-0.816)	0.068 (0.056-0.084)	0.251 (0.221-0.283)	0.979 (0.977-0.980)	0.101 (0.086-0.117)	0.144 (0.124-0.164)
24 hours	0.767 (0.757-0.776)	0.083 (0.072-0.095)	0.184 (0.165-0.206)	0.981 (0.979-0.982)	0.146 (0.128-0.165)	0.162 (0.145-0.183)

a Epic Deterioration Index data were available beginning July 1, 2020.

b Ensemble model predictions were generated only at time points when new pager messages were available.

Predictions from clinical pages and EDI were moderately correlated (Pearson’s R of 0.32, 0.31, and 0.30 for 24-, 12-, and 6-hour windows, respectively). The ensemble model, combining both data sources, consistently outperformed either model alone (Table 2; Appendix E), achieving its best performance 6 h before deterioration (AUROC [95% CI], 0.802 [0.77-0.821]). Within this 6-hour window, the ensemble detected 27% of events, compared to 18% detected by EDI. Within 24 h, detection rates were 18% vs 15%. Ensemble performance by deterioration type is summarized in Table 3. Discriminative ability was highest for cardiac arrest (AUROC 0.944-0.985) compared to Rapid Response (0.706-0.731) and ICU Transfer (0.793-0.843). Results across prediction thresholds are presented in Appendix F.

**Table 3.** Ensemble model classification performance by deterioration type.

	Score, no. (95% CI)					
	AUROC	AUPRC	Sensitivity	Specificity	PPV	F-Score
6 hours						
Rapid response	0.706 (0.656-0.746)	0.006 (0.004-0.009)	0.110 (0.054-0.170)	0.985 (0.984-0.986)	0.013 (0.006-0.019)	0.023 (0.011-0.035)
ICU transfer	0.843 (0.820-0.867)	0.028 (0.014-0.051)	0.231 (0.162-0.316)	0.985 (0.994-0.986)	0.028 (0.018-0.039)	0.050 (0.033-0.070)
Cardiac arrest	0.985 (0.960-0.991)	0.055 (0.036-0.091)	0.841 (0.712-0.917)	0.985 (0.984-0.986)	0.031 (0.026-0.037)	0.060 (0.050-0.072)
12 hours						
Rapid response	0.731 (0.700-0.760)	0.016 (0.011-0.022)	0.112 (0.069-0.168)	0.982 (0.981-0.983)	0.028 (0.016-0.041)	0.045 (0.027-0.065)
ICU transfer	0.835 (0.806-0.858)	0.041 (0.032-0.054)	0.268 (0.223-0.321)	0.982 (0.981-0.983)	0.053 (0.044-0.066)	0.089 (0.073-0.109)
Cardiac arrest	0.953 (0.917-0.978)	0.084 (0.055-0.146)	0.702 (0.614-0.821)	0.981 (0.980-0.982)	0.045 (0.036-0.056)	0.085 (0.067-0.105)
24 hours						
Rapid response	0.718 (0.705-0.734)	0.026 (0.022-0.031)	0.076 (0.053-0.107)	0.984 (0.983-0.985)	0.049 (0.030-0.060)	0.068 (0.038-0.077)
ICU transfer	0.793 (0.672-0.707)	0.038 (0.022-0.033)	0.183 (0.149-0.211)	0.984 (0.983-0.985)	0.069 (0.057-0.081)	0.100 (0.085-0.118)
Cardiac arrest	0.944 (0.919-0.960)	0.115 (0.073-0.195)	0.641 (0.552-0.727)	0.983 (0.982-0.984)	0.077 (0.063-0.092)	0.137 (0.113-0.163)

## Discussion

We developed a machine learning pipeline to predict clinical deterioration using pages sent among hospital teams. We found that predicting deterioration using only pages offered modest performance compared to the Epic Deterioration Index. However, we observed only moderate correlation between predictions from pages model and EDI, suggesting that each model captures distinct signals of deterioration. An ensemble approach that combined predictions from both models into a single score outperformed either model alone. These results suggest that clinical intuition is an important factor for identifying deterioration that is not well captured by existing early warning scores.

Clinicians often recognize signs of deterioration before empirical evidence is available through standard diagnostic tests or patient monitoring. Nurses, who interact directly and continuously with patients, are uniquely positioned to identify acute changes in condition. Prior research has identified qualitative indicators of clinical deterioration, including changes in mental status, agitation, difficulty speaking, shortness of breath, uncontrolled pain, and failure to respond to treatment.^20^^,^^43^^,^^44^ These bedside observations are not consistently documented in the EHR and rarely incorporated into existing EWS. Modern early warning scores incorporate a median of 12 predictors, commonly derived from structured data in the EHR.^16^ Prior research found that content discussed in clinical pages mention a mix of direct patient concerns and potentially concerning findings.^13^ We hypothesize that these insights, which are not routinely incorporated into EWS, contribute to the improved performance observed when combining predictions from our paging model with the EDI score.

Features of clinical intuition are inconsistently documented in the EHR, limiting their integration into early warning systems.^45^ Intuition and clinical reasoning are most often recorded in clinical notes. These notes offer robust information about a patient’s condition, but are often delayed from the time a clinician examines the patient and contain significant extraneous information and “copy forward” content, reducing their utility for predicting deterioration.^46^^,^^47^ Flowsheet entries and comments may also capture qualitative findings and concerns, but these data are also delayed and often do not reflect the patient’s current state, especially during busy or unpredictable shifts.^48^^,^^49^ In contrast, clinical pages are exchanged in real-time as part of routine workflow and serve as a primary means of communication among clinical teams.^27^ By systematically analyzing pager messages, our model aggregates collective intuition from multiple clinicians, capturing valuable insights that may never be formally documented in the EHR. Pages provide actionable insights into clinical intuition without increasing documentation burden or disrupting clinical workflow.

Our machine learning approach integrates predictions from clinical pages with existing EWS using a deployable architecture suited for clinical practice. We processed clinical pages using an LSTM-based model with a sliding window of the 10 most recent pages per encounter to provide relevant clinical context while minimizing outdated information. Each page was represented using BERT embeddings,^32^^,^^33^ which capture rich contextual features in clinical text while requiring minimal computational resources compared with modern large language models.

This work builds upon prior research which found that machine learning on all pages sent during an encounter accurately predicted clinical deterioration with AUROCs of 0.82-0.87.^13^ We found that modeling pages in real-time achieved only moderate discrimination (AUROC 0.63-0.72). We hypothesize that this difference in score may have been, in part, due to communications about end-of-encounter coordination which influenced predictions (eg, mentions of discharge or planning for end of hospitalization may suggest a lower likelihood of deterioration). However, we found that combining predictions from our clinical pages model with outputs from the EDI score using logistic regression significantly improved performance across all time horizons compared to the score from either model alone. Future prospective studies are needed to validate this enhanced predictive performance.

When evaluated at fixed decision thresholds, sensitivity was low across many prediction horizons for the composite deterioration outcome. This represents an important limitation of the current approach; higher sensitivity would be required for clinical deployment. Sensitivity was highest for cardiac arrest events, consistent with prior literature.^13^ We hypothesize that this may be because cardiac arrest is often preceded by more distinct and severe clinical signals, whereas rapid response activation and unplanned ICU transfer reflect more heterogeneous and context-dependent escalation decisions. We explored training separate models for each deterioration event type, but this did not result in meaningful or consistent performance improvements and would complicate potential future implementation. Accordingly, we focused on a single composite outcome and present these results as exploratory, highlighting the need for further methodological refinement and prospective evaluation.

Our study has several limitations. It was conducted at a single academic medical center using an Epic EHR, where paging is well integrated into clinical workflow. Findings may not generalize to other healthcare settings with different EHR systems, communication practices, or clinical workflows. Additionally, our study population primarily reflected the demographics of middle Tennessee, with a higher proportion of White individuals than the general U.S. population.^50^ This may introduce racial bias into our sample, and future work should validate these findings in diverse populations. This was a retrospective study. There may be additional confounding variables or aspects of clinical workflow that were not captured in our study. Future work should use a prospective randomized controlled trial to validate our study findings and validate clinical impact.

There is the potential for temporal bias related to the COVID-19 pandemic. Our training and test datasets span periods before and immediately after the onset of COVID-19, during which patient conditions, inpatient volumes, and staffing models may have changed. Although the training data included encounters from both pre- and post-pandemic periods, we did not explicitly assess differences in paging frequency or content across these time frames or rebalance training data by COVID era. Moreover, the holdout test set consisted of encounters occurring after June 2020, coinciding with the early phase of the pandemic, which may have influenced observed model performance. Future studies should explicitly examine temporal changes in paging behavior and evaluate model robustness across pre- and post-pandemic periods to better characterize and mitigate potential sources of bias.

It is also possible that some pages were not accurately linked to a patient and excluded from our study. Prior work has found that communication failures are common, including urgent or emergency pages being sent to the wrong physician^51^ or being linked to the wrong patient,^52^^,^^53^ which could impact model performance and subsequent patient care. More broadly, retrospective analysis of pager message content carries an inherent risk of residual information leakage, as messages may include explicit or implicit references to escalation decisions that occur close in time to deterioration events. While the use of a pre-event washout period reduces this risk, some leakage may persist for rapidly evolving scenarios. Prospective evaluation will be necessary to confirm that predictive signals arise from early clinical concern rather than documentation of escalation.

Because our pager-message model uses LSTM architectures with BERT embeddings, and the EDI score is proprietary, we were unable to identify specific features driving model predictions. Future research should explore approaches to characterize key predictors of deterioration from both structured EHR data and clinical communication signals. Finally, we used the Epic Deterioration Index (EDI) as a comparator because it is implemented in clinical practice, but it was not trained on our cohort. A de novo structured EHR-based model would provide a more direct comparison and could help disentangle the contributions of structured data vs pager-message data to predictive performance. In addition, a detailed, case-based error analysis comparing instances in which the ensemble model correctly identified deterioration that was missed by the EDI would be informative for understanding when communication-derived signals add the greatest value. Future work should also evaluate such models in parallel with pager-message models.

## Conclusion

Our study demonstrates that clinical pages contain valuable signals that are predictive of patient deterioration. We found that the patterns emerging in real-time communications between clinicians capture different aspects of deterioration than those detected by traditional early warning scores. By combining these signals, we achieved more robust detection of clinical deterioration than either method independently. This work validates the feasibility of leveraging clinical communications data and demonstrates the potential to improve existing early warning systems by incorporating these currently underutilized data streams. While further validation is needed, integrating real-time clinical communications with traditional early warning scores represents a promising approach to improve early detection of patient deterioration.

## Supplementary Material

ooag122_Supplementary_Data

## Data Availability

The data and code used in this study contain patient- and institution-level identifiers and cannot be shared publicly. We will make study materials, deidentified data, and deidentified code available upon reasonable request to investigators, in accordance with institutional policies.
