# Supplementary material for: Machine learning on asynchronous clinical pages to predict clinical deterioration
Source: JAMIA Open. 2026 Jul 17;9(4):ooag122. doi: 10.1093/jamiaopen/ooag122 (PMC13378451; doi:10.1093/jamiaopen/ooag122)
Supplement: ooag122_Supplementary_Data [file ooag122_supplementary_data.docx]

**Supplemental Material**

**Appendix A:** Example Clinical Pages

| 1 | BP 99/51 and pt getting bolus, do you want to hold metoprolol? thanks. |
| --- | --- |
| 2 | PTT is >200. Turn off heparin? |
| 3 | Pts temp is 38.6c despite tylenol. Cooling blanket started. neuro exam the same otherwise. 1s all the way around, withdrawls from pain in R upper, eyes opening only to pain. |
| 4 | HR has been in 120s for this pt since he has been back from TEE. Most recent BP 125/91. Sitting in chair. |
| 5 | BP 114/41 (62), resting in bed with husband at bedside. |
| 6 | wife noticed a bump on his R leg where he has gotten some of his vaccines. |
| 7 | Hi, our team ordered an amino acid screen on this pt for w/u of ALF(extensive neg w/u). Just wanted to hear your impression of result / see if consult is needed |
| 8 | Pt is meeting inpatient criteria. If agreed, please update status from observation to inpatient. Thanks |
| 9 | pt requesting PRN dilaudid for bilateral leg pain. he states Ultram does not help. |
| 10 | the 6MP dose is slightly out of the 10 percent range. still okay to give? thanks |

**Appendix B:** Hyperparameter Ranges

| **Parameter** | **Value Set** |
| --- | --- |
| Input Sequence Length | 64 |
| Batch Size | 256 |
| LSTM Layer 1 Units | {128 – 512, by 16} |
| LSTM Layer 2 Units | {128 – 512, by 16} |
| Dropout | {0.1, 0.2, 0.3, 0.4, 0.5} |
| Learning Rate | {1e-3, 1e-4, 5e-5, 1e-5} |
| Weight Decay Kernel | {0.0, 1e-5, 1e-4, 1e-3, 1e-2} |
| Weight Decay Bias | {0.0, 1e-5, 1e-4, 1e-3, 1e-2} |

**Appendix C:** Comparison of Bootstrapped Classification Metrics by Predicted Probability Cutpoint. Cutpoints were selected based on their proximity to the central cutpoint used to report model results, aiming for high sensitivity while preserving clinically meaningful positive predictive values.

| **Score, No. (95% CI)** | | |  |  | |  | |
| --- | --- | --- | --- | --- | --- | --- | --- |
| **Cutpoint** | **Sensitivity** | **Specificity** | | | **PPV** | | **F-Score** |
| **6-hours** |  |  | | |  | |  |
| 0.825 | 0.213 (.162-.258) | 0.962 (.961-.964) | | | 0.023 (.017-.029) | | 0.041 (.032-.052) |
| 0.85 | 0.194 (.151-.232) | 0.974 (.973-.975) | | | 0.030 (.023-.038) | | 0.052 (.040-.066) |
| 0.875 | 0.122 (.084-.158) | 0.983 (.982-.984) | | | 0.030 (.020-.040) | | 0.048 (.032-.065) |
| 0.90 | 0.104 (.067-.136) | 0.990 (.990-.991) | | | 0.043 (.028-.058) | | 0.061 (.039-.081) |
| 0.925 | 0.083 (.055-.112) | 0.995 (.995-.996) | | | 0.069 (.046-.091) | | 0.075 (.050-.100) |
| **12-hours** |  |  | | |  | |  |
| 0.675 | 0.182 (.148-.215) | 0.969 (.967-.970) | | | 0.052 (.042-.063) | | 0.081 (.066-.096) |
| 0.70 | 0.155 (.126-.186) | 0.975 (.973-.977) | | | 0.055 (0.045-.069) | | 0.082 (.066-.100) |
| 0.725 | 0.140 (.107-.172) | 0.980 (.979-.982) | | | 0.062 (.050-.077) | | 0.086 (.068-.105) |
| 0.75 | 0.111 (.085-.139) | 0.985 (.984-.986) | | | 0.065 (.050-.084) | | 0.082 (.063-.104) |
| 0.775 | 0.091 (.065-.121) | 0.989 (.988-.990) | | | 0.071 (.051-097) | | 0.079 (.058-.109) |
| **24-hours** |  |  | | |  | |  |
| 0.725 | 0.165 (.148-.184) | 0.968 (.967-.969) | | | 0.091 (.081-.103) | | 0.118 (.106-.131) |
| 0.75 | 0.134 (.116-.151) | 0.976 (.975-.977) | | | 0.099 (.086-.114) | | 0.114 (.099-.128) |
| 0.775 | 0.152 (.131-.173) | 0.963 (.961-.965) | | | 0.067 (.057-.079) | | 0.093 (.078-.109) |
| 0.80 | 0.079 (.066-.096) | 0.988 (.987-.989) | | | 0.117 (.097-.149) | | 0.094 (.078-.117) |
| 0.825 | 0.059 (.048-.074) | 0.992 (.992-.993) | | | 0.129 (.104-.164) | | 0.081 (.066-101) |

**Appendix D:** Encounter-Level Classification Performance. An encounter was classified as a true positive if: 1) the patient experienced clinical deterioration during their stay, and 2) the model made at least one correct prediction of deterioration within the specified time horizon (6, 12, or 24 hours) before the event, based on a page sent about the patient.

|  | **Score** | |  | |  | |  | |  | |
| --- | --- | --- | --- | --- | --- | --- | --- | --- | --- | --- |
|  | **AUROC** | **AUPRC** | | **Sensitivity** | | **Specificity** | | **PPV** | | **F-Score** |
| **Pages Model** |  |  | |  | |  | |  | |  |
| 6-hours | 0.697 | 0.041 | | 0.113 | | 0.957 | | 0.049 | | 0.069 |
| 12-hours | 0.732 | 0.059 | | 0.129 | | 0.952 | | 0.078 | | 0.097 |
| 24-hours | 0.704 | 0.085 | | 0.126 | | 0.959 | | 0.110 | | 0.117 |
| **EDI** |  |  | |  | |  | |  | |  |
| 6-hours | 0.726 | 0.053 | | 0.340 | | 0.870 | | 0.044 | | 0.078 |
| 12-hours | 0.740 | 0.095 | | 0.393 | | 0.865 | | 0.079 | | 0.132 |
| 24-hours | 0.748 | 0.127 | | 0.432 | | 0.866 | | 0.110 | | 0.176 |

**Appendix E:** Ensemble Model Performance for Out-of-Fold Predictions During Repeated Cross Validation Compared to EDI or Pages Alone. Confidence intervals were determined using 100 bootstrap samples.

|  |  | **Score, No. (95% CI)** | | |  |  | |  | |
| --- | --- | --- | --- | --- | --- | --- | --- | --- | --- |
| **Cutpoint** | **AUC** | **AUPRC** | **Sensitivity** | **Specificity** | | | **PPV** | | **F-Score** |
| **6-hours** |  |  |  |  |  |  |  |  |  |
| EDI (PF)* | 0.749  (0.731 - 0.762) | 0.022  (0.016 - 0.029) | 0.209  (0.185 - 0.239) | 0.966  (0.965 - 0.966) | | | 0.020  (0.018 - 0.024) | | 0.037  (0.032 - 0.043) |
| EDI (EF)* | 0.767  (0.731 - 0.802) | 0.004  (0.002 - 0.014) | 0.230  (0.166 - 0.294) | 0.957  (0.956 - 0.958) | | | 0.003  (0.002 - 0.004) | | 0.006  (0.004 - 0.008) |
| Pages | 0.711  (0.693 - 0.727) | 0.014  (0.011 - 0.019) | 0.066  (0.055 - 0.080) | 0.992  (0.992 - 0.993) | | | 0.028  (0.023 - 0.036) | | 0.039  (0.032 - 0.049) |
| Ensemble | 0.783  (0.779 - 0.786) | 0.024  (0.022 -0 .026) | 0.207  (0.200 - 0.214) | 0.980  (0.979 - 0.981) | | | 0.035  (0.034 - 0.036) | | 0.059  (0.058 - 0.061) |
| **12-hours** |  |  |  |  | | |  | |  |
| EDI (PF) | 0.752  (0.742 - 0.761) | 0.040  (0.035 - 0.046) | 0.217  (0.203 - 0.234) | 0.962  (0.962 - 0.963) | | | 0.045  (0.042 - 0.050) | | 0.075  (0.070 - 0.082) |
| EDI (EF) | 0.750  (0.732 - 0.771) | 0.013  (0.010 - 0.023) | 0.227  (0.200 - 0.255) | 0.952  (0.951 - 0.953) | | | 0.012  (0.010 - 0.014) | | 0.023  (0.019 - 0.026) |
| Pages | 0.733  (0.723 - 0.742) | 0.029  (0.026 - 0.035) | 0.173  (0.159 - 0.188) | 0.968  (0.967 - 0.968) | | | 0.043  (0.039 - 0.047) | | 0.068  (0.063 - 0.074) |
| Ensemble | 0.790  (0.788 - 0.792) | 0.048  (0.047- 0.050) | 0.196  (0.190 - 0.202) | 0.980  (0.979 - 0.981) | | | 0.076  (0.074 - 0.079) | | 0.109  (0.106 - 0.111) |
| **24-hours** |  |  |  |  | | |  | |  |
| EDI (PF) | 0.742  (0.733 - 0.749) | 0.063  (0.057 - 0.068) | 0.213  (0.200 - 0.223) | 0.961  (0.960 - 0.962) | | | 0.080  (0.075 - 0.085) | | 0.116  (0.109 - 0.122) |
| EDI (EF) | 0.731  (0.719 - 0.743) | 0.029  (0.024 - 0.034) | 0.218  (0.199 - 0.241) | 0.950  (0.949 - 0.951) | | | 0.031  (0.028 - 0.034) | | 0.054  (0.049 - 0.059) |
| Pages | 0.697  (0.691 - 0.705) | 0.047  (0.044 - 0.050) | 0.226  (0.216 - 0.240) | 0.944  (0.943 - 0.945) | | | 0.060  (0.056 - 0.064) | | 0.095  (0.090 - 0.101) |
| Ensemble | 0.766  (0.764 - 0.768) | 0.071  (0.069 - 0.073) | 0.166  (0.160 - 0.172) | 0.980  (0.979 - 0.981) | | | 0.117  (0.114 - 0.121) | | 0.137  (0.133 - 0.139) |

**PF: Page Frequency refers to an EDI prediction associated with each new page*

**EF: Epic EDI Frequency refers to an EDI prediction being made every 15 minutes*

**Appendix F:** Comparison of Bootstrapped Classification Metrics by Predicted Probability Cutpoint – Ensemble Model. Cutpoints were selected based on their proximity to the central cutpoint used to report model results, aiming for high sensitivity while preserving clinically meaningful positive predictive values.

| **Score, No. (95% CI)** | | |  |  | |  | |
| --- | --- | --- | --- | --- | --- | --- | --- |
| **Cutpoint** | **Sensitivity** | **Specificity** | | | **PPV** | | **F-Score** |
| **6-hours** |  |  | | |  | |  |
| 0.75 | 0.309 (.261-.352) | 0.973 (.971-.974) | | | 0.045 (.037-.052) | | 0.078 (.066-.091) |
| 0.775 | 0.282 (.234-.341) | 0.978 (.977-.979) | | | 0.050 (.041-.061) | | 0.086 (.069-.103) |
| 0.80 | 0.265 (.222-.313) | 0.983 (.982-.984) | | | 0.061 (.050-.072) | | 0.098 (.082-.170) |
| 0.825 | 0.244 (.200-.282) | 0.987 (.986-.988) | | | 0.070 (.057-0.085) | | 0.109 (.090-.131) |
| 0.85 | 0.209 (.175-.252) | 0.990 (.989-.991) | | | 0.080 (.065-.100) | | 0.116 (.095-.142) |
| **12-hours** |  |  | | |  | |  |
| 0.75 | 0.301 (.268-.334) | 0.968 (.966-.969) | | | 0.081 (.071-.095) | | 0.128 (.113-.148) |
| 0.775 | 0.277 (.243-.309) | 0.974 (.972-.975) | | | 0.091 (.077-.103) | | 0.137 (.118-.155) |
| 0.80 | 0.251 (.221-.283) | 0.979 (.977-.980) | | | 0.101 (.086-.117) | | 0.144 (.124-.164) |
| 0.825 | 0.216 (.183-.246) | 0.983 (.982-.985) | | | 0.109 (.091-.127) | | 0.145 (.121-.165) |
| 0.85 | 0.185 (.150-.216) | 0.987 (.986-.989) | | | 0.121 (.096-.144) | | 0.146 (.116-.171) |
| **24-hours** |  |  | | |  | |  |
| 0.725 | 0.238 (.218-.262) | 0.971 (.969-.972) | | | 0.127 (.114-.142) | | 0.165 (.150-.183) |
| 0.75 | 0.209 (.189-.234) | 0.976 (.975-.978) | | | 0.137 (.119-.157) | | 0.166 (.146-.187) |
| 0.775 | 0.184 (.165-.206) | 0.981 (.979-.982) | | | 0.146 (.128-.165) | | 0.164 (.145-.183) |
| 0.80 | 0.158 (.139-.179) | 0.985 (.984-.986) | | | 0.157 (.137-.182) | | 0.158 (.138-.181) |
| 0.825 | 0.135 (.118-.158) | 0.988 (.987-.989) | | | 0.170 (.143-.198) | | 0.151 (.132-.175) |
